# Adults with anomalous aortic origin of a coronary artery: impact of invasive functional testing on clinical decision making—insights from the MuSCAT registry

**DOI:** 10.1093/ehjimp/qyag010

**Published:** 2026-01-17

**Authors:** Diederick B H Verheijen, Anastasia D Egorova, Frank van der Kley, C J Koppel, Monique R M Jongbloed, Marcel A M Beijk, Robbert J de Winter, Michiel Voskuil, Michael G Dickinson, Peter Damman, Lodewijk J Wagenaar, Marco C Post, Dave R Koolbergen, Mark G Hazekamp, Hildo J Lamb, J Wouter Jukema, Philippine Kiès, Hubert W Vliegen

**Affiliations:** Center for Congenital Heart Disease Amsterdam-Leiden (CAHAL), Albinusdreef 2, Leiden 2333 ZA, The Netherlands; Department of Cardiology, Leiden University Medical Center, Albinusdreef 2, Leiden 2333 ZA, The Netherlands; Center for Congenital Heart Disease Amsterdam-Leiden (CAHAL), Albinusdreef 2, Leiden 2333 ZA, The Netherlands; Department of Cardiology, Leiden University Medical Center, Albinusdreef 2, Leiden 2333 ZA, The Netherlands; Center for Congenital Heart Disease Amsterdam-Leiden (CAHAL), Albinusdreef 2, Leiden 2333 ZA, The Netherlands; Department of Cardiology, Leiden University Medical Center, Albinusdreef 2, Leiden 2333 ZA, The Netherlands; Center for Congenital Heart Disease Amsterdam-Leiden (CAHAL), Albinusdreef 2, Leiden 2333 ZA, The Netherlands; Department of Cardiology, Leiden University Medical Center, Albinusdreef 2, Leiden 2333 ZA, The Netherlands; Center for Congenital Heart Disease Amsterdam-Leiden (CAHAL), Albinusdreef 2, Leiden 2333 ZA, The Netherlands; Department of Cardiology, Leiden University Medical Center, Albinusdreef 2, Leiden 2333 ZA, The Netherlands; Department of Anatomy & Embryology, Leiden University Medical Center, Leiden, The Netherlands; Center for Congenital Heart Disease Amsterdam-Leiden (CAHAL), Albinusdreef 2, Leiden 2333 ZA, The Netherlands; Department of Cardiology, Amsterdam University Medical Center, Amsterdam, The Netherlands; Center for Congenital Heart Disease Amsterdam-Leiden (CAHAL), Albinusdreef 2, Leiden 2333 ZA, The Netherlands; Department of Cardiology, Amsterdam University Medical Center, Amsterdam, The Netherlands; Department of Cardiology, University Medical Centre Utrecht, Utrecht, The Netherlands; Department of Cardiology, University Medical Centre Utrecht, Utrecht, The Netherlands; Department of Cardiology, Radboud University Medical Centre, Nijmegen, The Netherlands; Thorax Centre Twente, Medisch Spectrum Twente, Enschede, The Netherlands; Department of Cardiology, University Medical Centre Utrecht, Utrecht, The Netherlands; Department of Cardiology, St. Antonius Hospital, Nieuwegein, The Netherlands; Center for Congenital Heart Disease Amsterdam-Leiden (CAHAL), Albinusdreef 2, Leiden 2333 ZA, The Netherlands; Department of Cardiothoracic Surgery, Leiden University Medical Center, Leiden, The Netherlands; Center for Congenital Heart Disease Amsterdam-Leiden (CAHAL), Albinusdreef 2, Leiden 2333 ZA, The Netherlands; Department of Cardiothoracic Surgery, Leiden University Medical Center, Leiden, The Netherlands; Center for Congenital Heart Disease Amsterdam-Leiden (CAHAL), Albinusdreef 2, Leiden 2333 ZA, The Netherlands; Department of Radiology, Leiden University Medical Center, Leiden, The Netherlands; Department of Cardiology, Leiden University Medical Center, Albinusdreef 2, Leiden 2333 ZA, The Netherlands; Netherlands Heart Institute, Utrecht, The Netherlands; Center for Congenital Heart Disease Amsterdam-Leiden (CAHAL), Albinusdreef 2, Leiden 2333 ZA, The Netherlands; Department of Cardiology, Leiden University Medical Center, Albinusdreef 2, Leiden 2333 ZA, The Netherlands; Center for Congenital Heart Disease Amsterdam-Leiden (CAHAL), Albinusdreef 2, Leiden 2333 ZA, The Netherlands; Department of Cardiology, Leiden University Medical Center, Albinusdreef 2, Leiden 2333 ZA, The Netherlands

**Keywords:** anomalous aortic origin of the coronary artery, interarterial course, invasive functional testing, ischaemia, risk stratification

## Abstract

**Aims:**

In line with the contemporary European Society of Cardiology and American Heart Association/American College of Cardiology guidelines, the work-up for anomalous aortic origin of a coronary artery (AAOCA) should include anatomical and functional evaluation.

**Objective:**

This study aims to evaluate the impact of invasive functional testing vs. real-world non-invasive functional testing on the management strategy for adults with potentially malignant AAOCA.

**Methods and results:**

In this multicentre prospective study, all AAOCA patients, ≥16 years, with an interarterial or intraseptal course underwent diagnostic work-up, including anatomic and non-invasive functional evaluation. Invasive functional testing followed the MuSCAT protocol with fractional flow reserve (FFR) or instantaneous wave-free ratio (iFR)/resting full-cycle ratio (RFR), and intravascular ultrasound (IVUS) at baseline and during pharmacological stress. Seventy-six patients [53% female, median age 53 (44–59) years] were included. Sixteen (21%) initially presented with cardiac chest pain. High-risk anatomy, e.g. acute take-off angle <30°, was present in 75 (99%) patients. Non-invasive functional testing was positive in 10 (13%), negative in 59 (78%) and inconclusive in 2 (3%). Invasive functional testing showed discrepant results compared with non-invasive testing in 21 (28%) patients. In 15 (20% of the overall cohort) patients, the treatment recommendation was altered based on the invasive functional testing results.

**Conclusion:**

This study in adult AAOCA patients revealed a 28% discrepancy between non-invasive and invasive functional testing, altering management recommendations for 20% of the overall cohort. Implementing optimal functional testing modalities may enhance risk stratification for ischaemic events and sudden cardiac death, enabling tailored treatment strategies for AAOCA patients. Further prospective research with long-term follow-up is needed to evaluate clinical outcomes.

Registered in the Dutch Trial Register #NL8777; https://onderzoekmetmensen.nl/en.

## Graphical Abstract

**Graphical Abstract:**
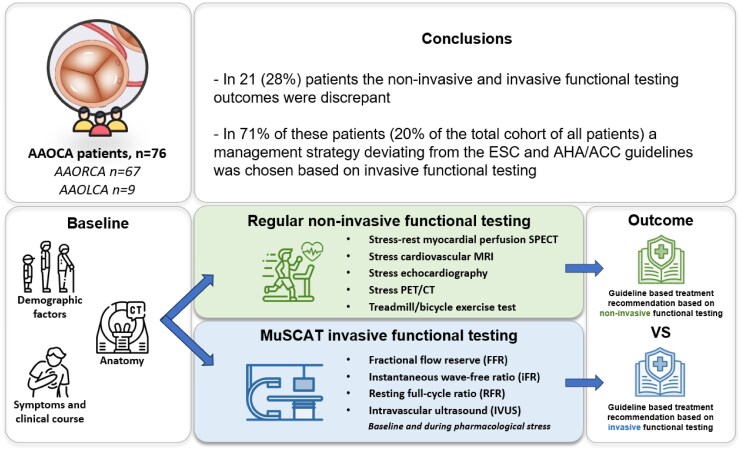
AAOCA, anomalous aortic origin of a coronary artery; AAOLCA, anomalous aortic origin of a left coronary artery; AAORCA, anomalous aortic origin of a right coronary artery; FFR, fractional flow reserve; iFR, instantaneous wave-free ratio; IVUS, intravascular ultrasound; MRI, magnetic resonance imaging; PET/CT, positron emission tomography/computed tomography; RFR, resting full-cycle ratio; SPECT, single-photon emission computerized tomography.

## Introduction

Anomalous aortic origin of a coronary artery (AAOCA) is a congenital heart defect with a prevalence of 0.1–1% in the general population.^1–3^ AAOCA patients, especially those at an age of under 35 years old or professional athletes, are at an increased risk for sudden cardiac death (SCD), primarily during or immediately after vigorous exercise.^4,5^ In patients above 35 years, the risk of SCD attributable to AAOCA declines, likely due to a combination of factors, including the progressive reduction in physical activity with age and the age-related decrease in peak heart rate at maximal exercise capacity, which collectively results in a lower myocardial oxygen demand.^6–12^ Additionally, age-associated reductions in arterial compliance may further decrease the risk of external compression of the interarterial segment of the AAOCA between the aorta and pulmonary artery.^13^ However, SCD is still reported in selected cases, highlighting the need for better risk-stratification.^14–16^ Symptoms also do not appear indicative for risk of SCD, as prior studies have shown a very limited correlation between clinical symptoms and the haemodynamic significance of AAOCA.^17^

Therefore, a comprehensive cardiac work-up is generally indicated to stratify for the risk of an ischaemia or SCD. Both contemporary European Society of Cardiology (ESC) and American Heart Association/American College of Cardiology (AHA/ACC) guidelines incorporate anatomical and non-pharmacological functional stress imaging studies in their recommendations for diagnostic work-up of AAOCA patients.^18,19^ Regarding anatomical imaging studies, previous research has demonstrated that high-risk anatomy may be present even in the absence of positive findings from both non-invasive and invasive functional testing.^20^ This supports the notion that deemed high-risk anatomy does not necessarily result in significant coronary flow obstruction, warranting additional testing for the individual risk assessment.

Recently, the AAOCA-EURO registry reported that diagnostic protocols, especially for functional evaluation, predominantly include non-invasive testing and are in practice highly heterogenous.^21^ This heterogeneity encompasses the use of adenosine and regadenoson during pharmacological stress, despite non-pharmacological stress being recommended by the guidelines.^18,19^ In situations where conventional physical stress cardiac imaging is unavailable or inadequate, pharmacological stress using dobutamine or adrenaline is preferred, which more accurately mimics the haemodynamic changes that occur during exertion, in contrast to the hyperaemia induced by adenosine or regadenoson.^22^ An accompanying factor enhancing heterogeneity is that non-invasive functional testing modalities do not evaluate all (patho)physiologically relevant aspects of AAOCA.^22,23^

Invasive functional testing appears to overcome some of these limitations. Over the past decade invasive functional testing has become an established technique in evaluating ischaemia in patients with epicardial coronary artery disease as well as those with ischaemia with non-obstructed coronary arteries. Invasive functional testing using fractional flow reserve (FFR), instantaneous-wave free ratio (iFR), resting full-cycle ratio (RFR) and/or intravascular ultrasound (IVUS), have demonstrated their reproducibility in guiding the management of patients with (non-)obstructive coronary artery disease.^20,24–27^ However, these techniques have to date not systematically been evaluated in AAOCA patients. To address this gap, these modalities were systematically included in the diagnostic work-up protocol for AAOCA in the first Multicenter Study on Coronary Anomalies in The Netherlands—the MuSCAT study.^27^ Invasive functional testing, with its high spatial and temporal resolution, can assess both the fixed and dynamic components of AAOCA when combined with adequate pharmacological stress.^22,28^

Enhanced understanding of these components may lead to improved risk stratification for ischaemia and SCD and facilitate the development of personalized treatment strategies for AAOCA patients.

The aim of this study is to evaluate the impact of invasive functional testing compared with regular non-invasive functional testing on the management strategy for adults with an AAOCA with a potentially malignant course.

## Methods

In this national prospective multicentre study on coronary anomalies in the Netherlands: MuSCAT (registered in the Dutch Trial Register #NL8777; https://onderzoekmetmensen.nl/en) all consecutive patients of 16 years and older with potentially malignant AAOCA in whom the diagnostic work-up was performed between January 2021 and January 2024 in six participating centres were eligible for inclusion,^27^  Supplementary data online, *Figure S1*. A potentially malignant AAOCA was defined as an AAOCA with an interarterial or intraseptal course as accessed on computed tomography angiography (CTA), in line with the 2020 ESC Guidelines.^27^ The diagnostic work-up included standard of care AAOCA work-up with evaluation of symptoms by a dedicated adult congenital heart disease (ACHD) cardiologist, electrocardiogram (ECG), transthoracic echocardiography (TTE), CTA, and non-invasive functional testing. Additional MuSCAT AAOCA work-up included coronary angiography (CAG) with invasive functional testing, e.g. FFR, instantaneous wave-free ratio (iFR), RFR, and/or IVUS, both at baseline and at pharmacologic stress.^27^ Exclusion criteria were the presence of a concomitant congenital heart defect or significant obstructive coronary artery disease in the AAOCA effluence.

Demographic and clinical data were extracted from electronic health record systems of the participating sites (EPD-Vision©, Leiden University Medical Center, Leiden, the Netherlands; HiX, Chipsoft, Amsterdam, the Netherlands; and Epic, Epic Systems, Verona, WA, USA). Angina was classified as cardiac chest pain, possibly cardiac chest pain or likely non-cardiac chest pain in accordance with the 2023 ESC Guidelines for the management of acute coronary syndromes.^29^

The management strategy was determined by consensus within the ACHD heart team, which was comprised of ACHD cardiologists, dedicated interventional cardiologists and cardiothoracic surgeons specialized in AAOCA.

For comparison of management strategies based on non-invasive and invasive functional testing, the 2020 ESC and 2018 AHA/ACC guidelines dictating the management of AAOCA were used as the gold standard of care. There were three subgroups of AAOCA patients who are not defined in the guidelines, yet are encountered in practice: (1) symptomatic (but other than typical angina or cardiac chest pain) patients with high-risk anatomy and proven ischaemia, (2) asymptomatic patients with an anomalous aortic origin of a right coronary artery (AAO**R**CA) with high-risk anatomy, but without proven ischaemia, and (3) asymptomatic patients with anomalous aortic origin of a left coronary artery (AAO**L**CA) ≥ 35 years of age without proven ischaemia and without high-risk anatomy. These three endotypes were defined and used in this study for stratification and analysis purposes.

### Computed tomography angiography

CTA images were evaluated as previously described blinded to clinical status and the decision making by an experienced investigator.^20^ Coronary anatomy was described according to the Leiden Convention coronary coding system,^30^  Supplementary data online, *Figure S2*. An interluminal space (ILS) of <0.95 mm at 2 mm from the coronary ostium defined presence of an intramural course and distance from the coronary ostium to an ILS ≥0.95 mm defined the length of the intramural segment.^31^ High-risk anatomy was defined as the presence of ≥1 high-risk anatomical features, including features such as an intramural course and orifice anomalies (slit-like orifice, acute-angle take-off, orifice >1 cm above the sinotubular junction), in accordance with the current ESC guidelines.^18^ Coronary artery disease was reported according to the Coronary Artery Disease Reporting and Data System.^32^

### Non-invasive functional testing

All patients underwent non-invasive functional testing. The methods selected for non-invasive functional testing were determined by local practices and expertise. Procedures for protocols, stress, and image interpretation followed international guidelines and consensus documents.^33–37^

### Invasive functional testing

All patients underwent coronary angiography with invasive functional testing: FFR, iFR, RFR, and/or IVUS, Supplementary data online, *Table S1*. The coronary angiogram was exclusively used for assessment of significant obstructive coronary artery disease. Visual lumen reduction on coronary angiography of ≥50% in the left main coronary artery or ≥70% in a major coronary artery was considered significant.^38^

Invasive functional testing was performed with FFR, iFR, RFR, and/or IVUS at baseline and during pharmacological stress.

FFR, iFR, and/or RFR measurements at baseline assessed coronary flow obstruction and IVUS at baseline assessed the anatomical shape of the coronary artery. These measurements collectively provided an evaluation of the fixed components of the AAOCA. Baseline measurements were obtained with iFR, RFR, and/or IVUS after an intracoronary bolus of nitroglycerin (0.2 mg i.c.) and with FFR during adenosine infusion (140 µg/min/kg i.v.), as previously described.^27^

FFR, iFR, and/or RFR measurements during pharmacological stress assessed both the increase in coronary flow obstruction relative to the baseline measurement and the absolute level of coronary flow obstruction during stress. IVUS during pharmacological stress assessed anatomical changes, such as the consequences of any (dynamic) external compression. These measurements collectively provided an evaluation of the haemodynamical stress-related, also referred to as the dynamic, components of the AAOCA. FFR, iFR, RFR, and/or IVUS measurements during pharmacological stress were performed with dobutamine (up to 40 µg/kg/min) or after an intravenous bolus of adrenaline (0.025–0.1 mg i.v.).^39^ Measurements during pharmacological stress were considered conclusive if the target heart rate of >130 bpm or target systolic blood pressure >150 mmHg was reached, in accordance with the literature.^39^

The methods selected for invasive functional testing were determined by local practices and expertise, Supplementary data online, *Table S1*. Invasive functional testing was considered positive if the cut-off values for FFR, iFR, RFR, or IVUS were met.^40,41^ Dynamic compression was assessed with IVUS during pharmacological stress by the primary operator.^20^ Cut-off values used for a positive invasive functional test are provided in Supplementary data online, *Table S1*.

### Ethics statement

The Multicenter Study on Coronary Anomalies in The Netherlands: MuSCAT is a national, prospective, multicentre study with a structured care pathway to assess an evidence-based diagnostic and treatment approach in AAOCA patients.^27^ All tests and procedures performed involving human participants were in accordance with the ethical standards of the institutional and/or national research committee and with the 2013 Helsinki declaration or comparable ethical standards. Appropriate local scientific board approval, as well as approval from the Medical Ethics Committee Leiden-the Hague-Delft (reference number P19.089) was obtained. All patients provided written consent for registration, analysis, and publication of their data.

### Statistical analysis

Statistical analysis was performed using SPSS (version 25; SPSS Inc., Chicago, IL, USA) and R statistical software (version 4.3.1, R Foundation for Statistical Computing, Vienna, Austria). Categorical data were presented as numbers and percentages. Normal distribution of continuous data was assessed visually and using the Shapiro–Wilk test. Normally distributed continuous data were presented as mean ± standard deviation.

## Results

**Figure 1:**
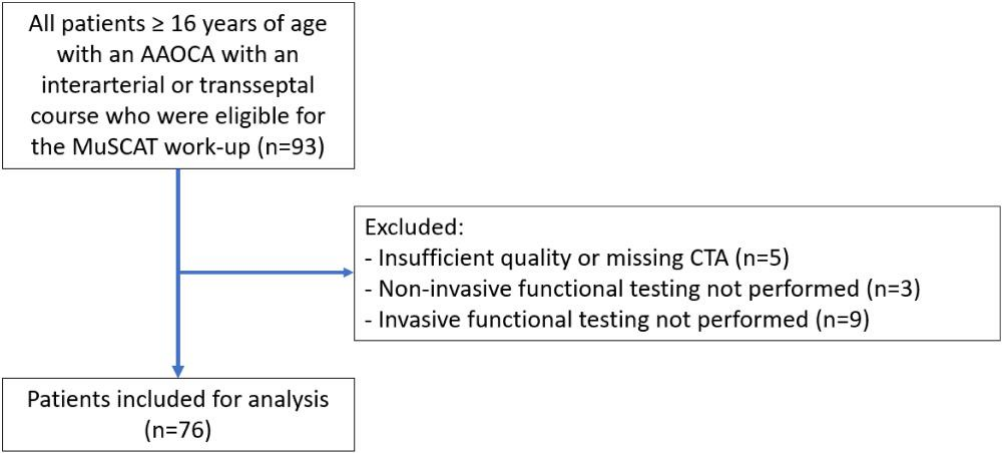
Patients included for analysis.

Ninety-three patients were eligible for inclusion, *Figure 1*. Seventeen patients were excluded from analysis due to incomplete diagnostic work-up [insufficient quality of the CTA (*n* = 5), non-invasive functional testing missing (*n* = 3), invasive functional testing missing (*n* = 9)]. The remaining 76 patients [53% female, median age at AAOCA diagnosis 53 (44–59) years] were included for analysis. Baseline characteristics of included patients are shown in *Table 1*.

**Table 1:** Baseline characteristics

	Patients *n* = 76
Female, *n* (%)	40 (53)
Age first diagnosis AAOCA (years), median [IQR]	53 [44–59]
BMI (kg/m^2^), mean ± SD	28.0 ± 4.9
History, *n* (%)	
Hypertension	31 (41)
Myocardial infarction	1 (1)
Heart failure	2 (3)
Arrhythmia	
Atrial fibrillation or atrial flutter	3 (4)
Other supraventricular arrhythmia	3 (4)
Premature ventricular contractions	5 (7)
Non-sustained ventricular tachycardia	2 (3)
Sustained ventricular tachycardia	0
Valvular heart disease	5 (7)
Cerebrovascular accident/TIA	6 (8)
Peripheral artery disease	5 (7)
COPD/asthma	12 (16)
Diabetes mellitus	11 (15)
Hypercholesterolaemia	22 (29)
History of smoking or current smoking	26 (35)
Family history of cardiovascular disease	21 (28)
Medication at first presentation, *n* (%)	
Beta-blocker	13 (17)
ACEI/ARB	24 (32)
CCB	11 (15)
Antiplatelet	10 (13)
Anticoagulant	2 (3)
Statin	16 (21)
Other cholesterol lowering medications	3 (4)
None	24 (32)
Symptoms at 1st presentation MuSCAT centre, *n* (%)	
Cardiac chest pain	16 (21)
Possibly cardiac chest pain	23 (30)
Likely non-cardiac chest pain	14 (18)
Dyspnoea	14 (18)
Palpitations	8 (11)
(Pre-)syncope	1 (1)
None related to AAOCA/incidental finding	13 (17)
Laboratory tests	
eGFR (mL/min 1.73 m^2^), *n* (%)	
≥60	73 (96)
≥30–60	3 (4)
<30	0
Electrocardiogram	
Rhythm	58 (76)
Sinus rhythm	16 (21)
Sinus bradycardia	2 (3)
Supraventricular tachycardia	
Heart axis, *n* (%)	
Intermediate	74 (97)
Left	1 (1)
Right	1 (1)
QRS duration, *n* (%)	
Normal (QRS <100 ms)	48 (63)
Interventricular conduction delay (QRS 100–120 ms)	23 (30)
Complete bundle branch block (QRS >120 ms)	5 (7)
ST-segment elevation or depression, *n* (%)	0
Repolarization abnormalities, *n* (%)	8 (11)
Transthoracic echocardiography, *n* (%)	
Normal LV function	66 (92)
Normal RV function	71 (99)
Coronary arteries visible	2 (3)

AAOCA, anomalous aortic origin of a coronary artery; ACEI, ACE-inhibitor; ARB, angiotensin receptor blocker; BMI, body mass index; CCB, calcium channel blocker; LV, left ventricular; TTE, transthoracic echocardiography.

One (1%) patient had a history of myocardial infarction. Prevalence of cardiovascular risk factors were hypertension in 31 (41%) patients, diabetes mellitus in 11 (15%) patients, hypercholesterolaemia in 22 (29%) patients, history of smoking or active smoking status in 26 (35%) patients, and family history of cardiovascular disease in 21 (28%) patients. Normal systolic left ventricular (LV) function and right ventricular function were present in 66 (92%) and 71 (99%) patients, respectively.

Symptoms at time of initial presentation were: cardiac chest pain in 16 (21%) patients, possibly cardiac chest pain in 23 (30%) patients, likely non-cardiac chest pain in 14 (18%) patients, dyspnoea in 14 (18%) patients, palpitations in 8 (11%) patients, (pre-)syncope in 1 (1%) patient, and non-AAOCA related complaints or asymptomatic (incidental finding) in 13 (17%) patients, *Table 1*.

### Computed tomography angiography

AAORCA was present in 67 (88%) patients, all with an interarterial course, *Table 2*. AAOLCA was present in 9 (12%) patients; 5 (56%) with an interarterial and 4 (44%) with an intraseptal course. In 56 (74%) patients, the AAOCA was the dominant coronary artery. High-risk anatomy was present in all AAORCA and in 8 (89%) AAOLCA patients: take-off above the sinotubular junction in 17 (22%), take-off above the pulmonary valve in 20 (26%), a slit-like orifice in 48 (63%), acute coronary take-off angle (<30°) in 67 (88%), and a suspicion of an intramural course (based on ILS <0.95 mm at 2 mm from ostium) in 52 (68%) patients. Median length of the intramural course in patients with a suspected intramural course was 7.3 [2.7–9.2] mm. No significant obstructive epicardial coronary artery disease (CADRADS ≤ 3) was present in any of the patients at baseline, as determined by CTA, which was confirmed by coronary angiography.

**Table 2:** Computed tomography angiography characteristics

	Patients *n* = 76
Anatomy according to Leiden convention, *n* (%)	
AAORCA	
2R*,LCx	65 (86)
2R*,L,Cx	1 (1)
2R*LCx	1 (1)
AAOLCA	
1R,LCx*	3 (4)
1RLCx*	2 (3)
1R,L-2Cx	1 (1)
1RLCx	1 (1)
1R,LCx	2 (3)
Interarterial course, *n* (%)	72 (95)
Intraseptal course, *n* (%)	4 (5)
Cardiac dominant AAOCA, *n* (%)	
AAORCA	56 (74)
AAOLCA	0
Presence of ≥1 high-risk anatomical feature,^a^ *n* (%)	
AAORCA	67 (100)
AAOLCA	8 (89)
Take-off in relation to STJ, *n* (%)	
Above	17 (22)
At the level of	31 (41)
Below	28 (37)
Take-off in relation to PuV, *n* (%)	
Above	20 (26)
At the level of	42 (55)
Below	14 (18)
Orifice shape, *n* (%)	
Slit-like	48 (63)
Oval	25 (32)
Round	3 (4)
Coronary take-off angle <45°, *n* (%)	76 (100)
Coronary take-off angle <30°, *n* (%)	67 (88)
Proximal narrowing, *n* (%)	41 (54)
Intramural course	
Suspicion of an intramural course (ILS <0.95 mm at 2 mm from ostium), *n* (%)	52 (68)
Length of intramural course if suspected (distance in mm from ostium to ILS >0.95 mm), median [IQR]	7.3 [2.7–9.2]
Coronary triangulated orifice area (CTOA), median [IQR]	3.4 [2.0–5.0]
Degree of CAD in AAOCA	
None	60 (79)
1–24%	13 (17)
24–49%	3 (4)
Overall CADRADS	
0	46 (61)
1	22 (29)
2	7 (9)
3	1 (1)

^a^As referred to in the 2020 ESC Guidelines for the management of adult congenital heart disease, including features such as an intramural course and orifice anomalies (slit-like orifice, acute-angle take-off, orifice >1 cm above the sinotubular junction).

AAOCA, anomalous aortic origin of a coronary artery; AAOLCA, anomalous aortic origin of a left coronary artery; AAORCA, anomalous aortic origin of a right coronary artery; CAD, coronary artery disease; CADRADS, Coronary Artery Disease Reporting and Data System; CTOA, coronary triangulated orifice area; ILS, interluminal space; PuV, pulmonary valve; STJ, sinotubular junction.

### Non-invasive functional testing

Non-invasive functional testing was performed using physical exercise or dobutamine in 71 (93%) patients and with adenosine or regadenoson in 5 (7%) patients, *Table 3*. Non-invasive functional testing was positive in 10 (13%) patients, negative in 63 (83%) patients and inconclusive in 3 (4%) patients.

**Table 3:** Non-invasive and invasive functional testing modalities and outcomes

	Patients, *n* = 76
Non-invasive functional testing, *n* (%)	
SPECT, physical exercise	51 (67)
SPECT, regadenoson	2 (3)
SPECT, adenosine	3 (4)
SPECT, dobutamine	2 (3)
PET/CT, dobutamine	4 (5)
MRI, dobutamine	4 (5)
TTE, dobutamine	3 (4)
TTE, physical exercise	1 (1)
Cycle ergometry	6 (8)
Result non-invasive functional testing, *n* (%)	
Positive	10 (13)
Negative	63 (83)
Inconclusive	3 (4)
Invasive functional testing, *n* (%)	
FFR adenosine	50 (66)
iFR baseline	54 (71)
RFR baseline	10 (13)
IVUS baseline	52 (68)
FFR adrenaline/dobutamine	44 (57)
iFR adrenaline/dobutamine	53 (70)
RFR adrenaline/dobutamine	1 (1)
IVUS adrenaline/dobutamine	36 (47)
Result invasive functional testing, *n* (%)	
Positive	15 (20)
Negative	59 (78)
Inconclusive	2 (3)
Dynamic compression on IVUS	
Significant	9 (17)
Not significant	41 (79)
Inconclusive	2 (4)

FFR, fractional flow reserve; iFR, instantaneous wave-free ratio; IVUS, intravascular ultrasound; MRI, magnetic resonance imaging; PET/CT, positron emission tomography/computed tomography; RFR, resting full-cycle ratio; SPECT, single-photon emission computerized tomography; TTE, transthoracic echocardiography.

### Invasive functional testing

**Figure 2:**
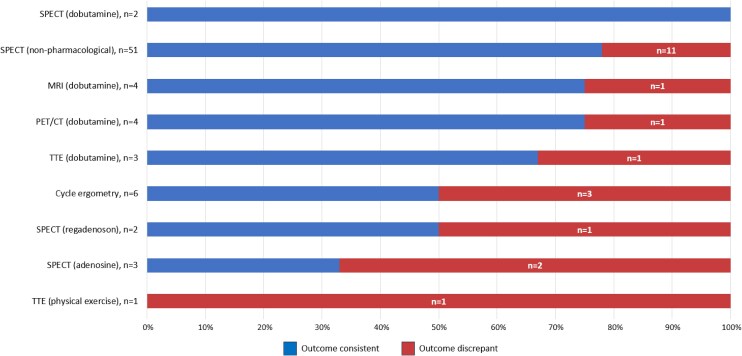
Bar chart illustrating the non-invasive functional testing modalities (*Y*-axis) and the percentage of congruent outcomes between the non-invasive and invasive functional testing (blue bar). The number in the red bar illustrates the amount of tests with a discrepant outcome between the non-invasive and invasive functional testing (red). Abbreviations: MRI, magnetic resonance imaging; PET/CT, positron emission tomography/computed tomography; SPECT, single-photon emission computerized tomography; TTE, transthoracic echocardiography.

Invasive functional testing with FFR, iFR, and/or RFR was performed in all and with IVUS in 52 (68%) patients. Adrenaline was administered during invasive functional testing in 36 (47%) patients, whereas dobutamine was used in 25 (33%) patients. Invasive functional testing was positive in 15 (20%) patients, negative in 59 (78%) patients and inconclusive in 2 (3%) patients. Dynamic compression with IVUS was significant in 9 (17%) patients, non-significant in 41 (79%) patients, and inconclusive in 2 (4%) patients. In 21 (28%) patients the non-invasive and invasive functional testing outcomes were discrepant, *Figure 2*. Overlap of positive and negative findings for high-risk coronary anatomy, non-invasive functional testing, invasive functional assessment (FFR/iFR/RFR), and external compression on IVUS are shown in Supplementary data online, *Figure S3*. Of the 10 (13%) patients with a positive non-invasive functional test [physical exercise single-photon emission computed tomography (SPECT) in 6 (60%) patients, adenosine SPECT in 2 (20%) patients, and cycle ergometry in 2 (20%) patients], invasive functional testing was negative in 8 (80%) patients, non-conclusive in 1 (10%) patients, and only (congruently) positive in 1 (10%) patient, Supplementary data online, *Figure S4*. Of the 63 (83%) patients with negative non-invasive functional testing, invasive functional testing was negative in 49 (78%) patients, positive in 13 (21%) patients, and non-conclusive in 1 (2%) patient, Supplementary data online, *Figure S4*.

### Impact of invasive functional testing on guideline recommendations

**Figure 3:**
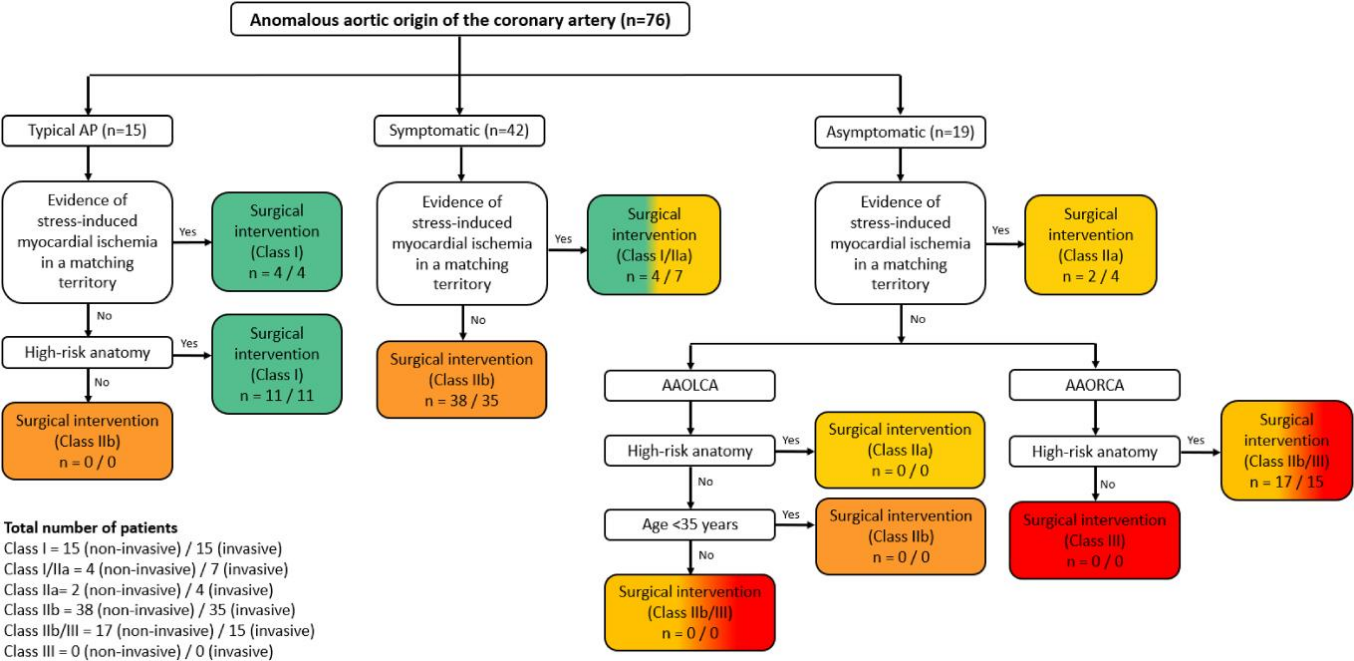
Flow diagram resulting in ESC class of recommendation^18^ and currently missing categories of AAOCA patients with a class of recommendation for surgical therapy with the number of patients per category included in this study based on non-invasive functional testing and based on invasive functional testing. Number of patients per category are reported as ‘*n* = “based on non-invasive functional testing”/“based on invasive functional testing”’. AAOLCA, anomalous aortic origin of a left coronary artery; AAORCA, anomalous aortic origin of a right coronary artery; AP, angina pectoris.

**Figure 4:**
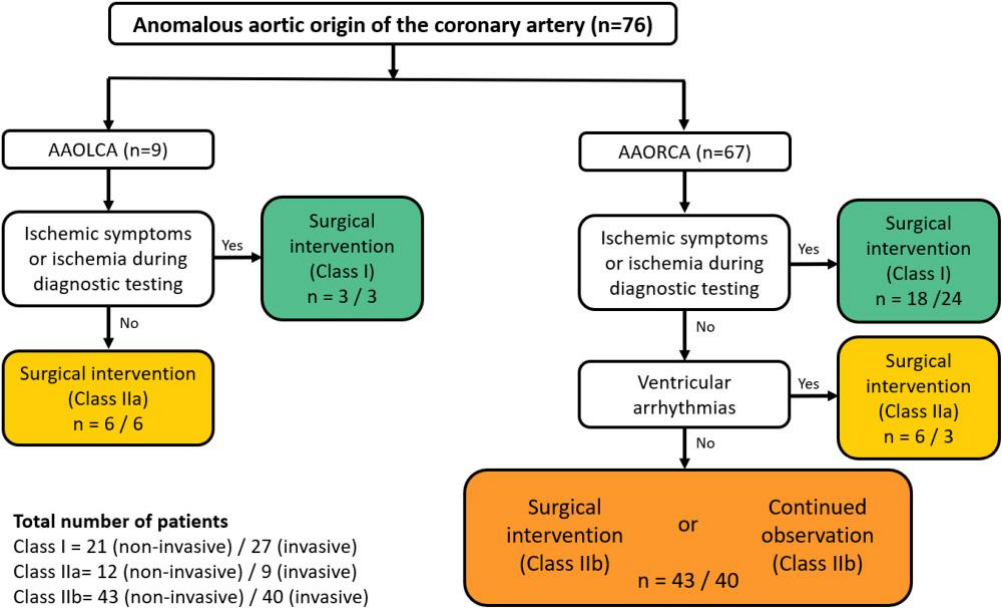
Flow diagram, modified from the AHA/ACC guidelines,^19^ resulting in AHA/ACC class of recommendation for surgical therapy with the number of patients per category included in this study based on non-invasive functional testing and based on invasive functional testing. Number of patients per category are reported as ‘*n* = “based on non-invasive functional testing”/“based on invasive functional testing”’.

*Figures 3* and *4* present flow diagrams illustrating the ESC and AHA/ACC guideline recommendations for the management of AAOCA, including the number of patients per class of recommendation based on non-invasive functional testing and the evolving insight after considering invasive functional testing. The number of patients with a class I, I/IIa, or IIa recommendation for surgical intervention increased with 5 (7%) based on the ESC guideline and with 3 (4%) based on the AHA/ACC guidelines.

**Figure 5:**
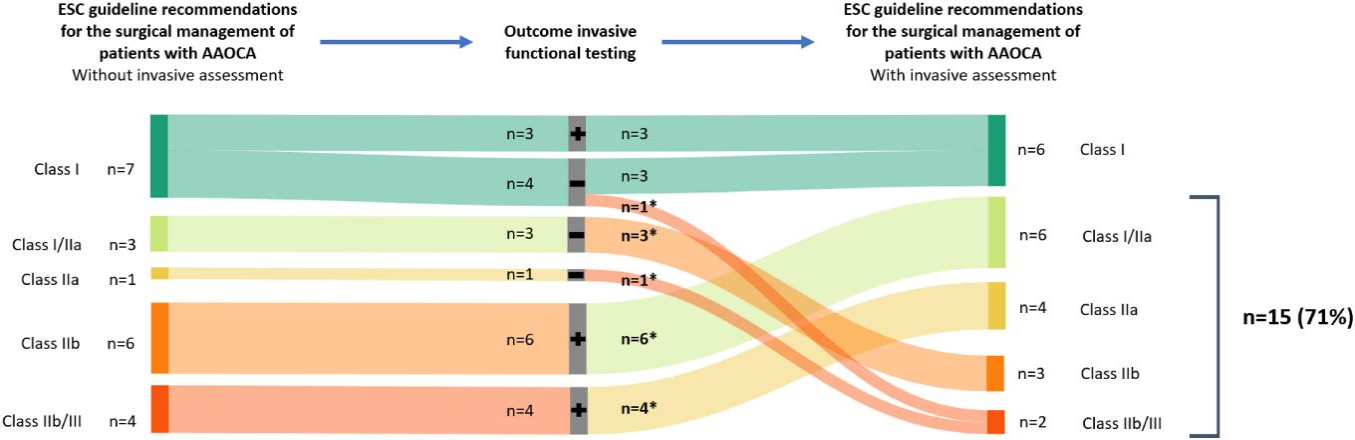
Sankey diagram illustrating the ESC guideline^18^ class of recommendation for surgical therapy based on the work-up without the invasive functional testing (left), the outcomes of the invasive functional testing (middle) and the ESC guideline class of recommendation for surgical therapy based on the work-up with the invasive functional testing (right) of all patients in whom the outcomes of the non-invasive functional testing was discrepant to the outcome of the invasive functional testing (*n* = 21). Outcome invasive functional testing; **+**: Positive invasive functional testing, negative non-invasive functional testing; **−**: Negative invasive functional testing, positive non-invasive functional testing. * Class of recommendation for surgical therapy was changed based on the outcome of the invasive functional testing. Abbreviations: AAOCA, anomalous aortic origin of a coronary artery; ESC, European Society of Cardiology.

**Figure 6:**
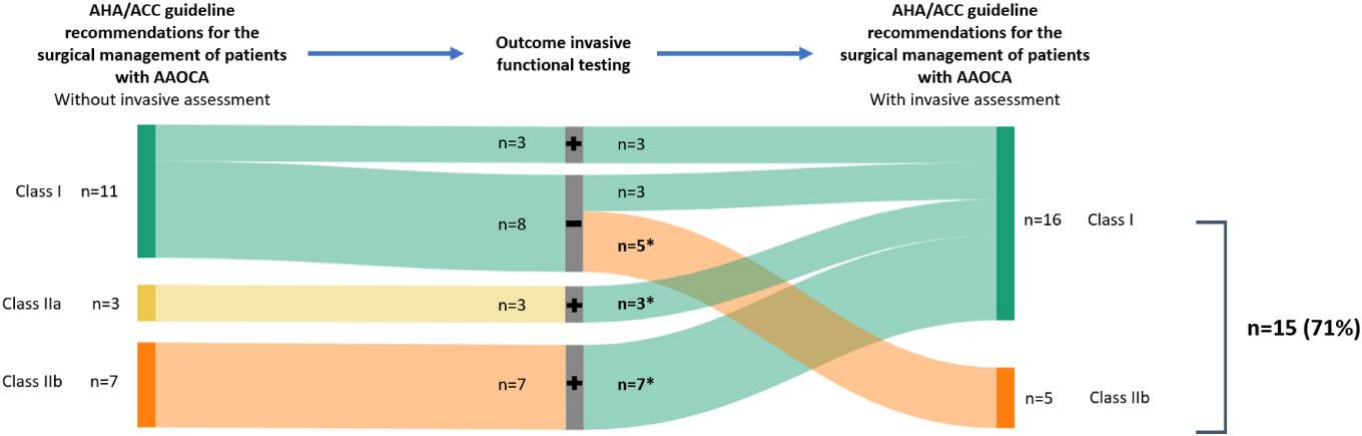
Sankey diagram illustrating the AHA/ACC guideline^19^ class of recommendation for surgical therapy based on the work-up without the invasive functional testing (left), the outcomes of the invasive functional testing (middle) and AHA/ACC guideline class of recommendation for surgical therapy based on the work-up with the invasive functional testing (right) of all patients in whom the outcomes of the non-invasive functional testing was discrepant to the outcome of the invasive functional testing (*n* = 21). Outcome invasive functional testing; **+**: Positive invasive functional testing, negative non-invasive functional testing; **−**: Negative invasive functional testing, positive non-invasive functional testing. *Class of recommendation for surgical therapy was changed based on the outcome of the invasive functional testing. Abbreviations: AAOCA, anomalous aortic origin of a coronary artery; AHA/ACC, The American Heart Association/The American College of Cardiology.

*Figures 5* and *6* demonstrate the individual impact of the invasive functional testing outcomes of the 21 patients with discrepant non-invasive and invasive functional testing outcomes on the recommendations for surgical treatment according to the ESC and AHA/ACC guidelines. Of the 21 patients with discrepant non-invasive and invasive functional testing outcomes, the class of recommendations for surgical treatment was altered in 15 (71%) compared with the current ESC guideline (*Figure 5*). Of these 15 patients, in 5 (33%) patients a recommendation for surgical treatment (class I, I/IIa, or IIa) based on non-invasive functional testing changed to a recommendation for conservative treatment (class IIb, IIb/III, or III) and in 10 (67%) patients vice versa. In 6 (29%) patients, the treatment recommendations for surgical treatment did not change. Treatment recommendation in these patients was based on typical AP and high-risk anatomy, resulting in a class I recommendation for surgical therapy despite negative invasive functional testing. Based on the AHA/ACC guideline (*Figure 6*), of the 21 patients with discrepant non-invasive and invasive functional testing outcomes, class of recommendation was altered in 15 (71%). In 5 (24%) patients, treatment recommendation changed from class I to class IIb and in 7 (33%) patients, treatment recommendation changed from class IIb to class I, significantly altering treatment recommendation.

### Impact of invasive functional testing on the management strategy

In the 74 (97%) patients with a conclusive outcome of invasive functional testing, a positive outcome resulted in surgical therapy and a negative outcome in a conservative treatment in 65 (88%) patients. Of the 59 patients with negative invasive functional testing [non-invasive testing outcomes; negative 49 (83%), positive 8 (14%), and non-conclusive 2 (3%)], 53 (90%) were managed conservatively. Six (10%) patients underwent surgery for AAOCA despite negative invasive functional testing [non-invasive testing outcomes; negative 3 (50%), positive 3 (50%)]. Indication for surgery in these patients was based on (clinical) characteristics: persistent cardiac chest pain in four (67%) patients and additional characteristics, such as vigorous exercise activity, in two (33%) patients. Of the 15 patients with positive invasive functional testing, 12 (80%) had surgical therapy and 3 (20%) were managed conservatively. Two (67%) of these three patients were treated conservatively because they were asymptomatic and one (33%) patient was treated conservatively after shared decision making, due to an increased surgical risk associated with a BMI of 35 kg/m^2^.

## Discussion

The first prospective multicentre study on coronary anomalies in the Netherlands (MuSCAT study) evaluated the impact of invasive functional testing compared with regularly used non-invasive functional testing on the management strategy for adults with an AAOCA with a potentially malignant course. The main findings of this study are: (1) there is a 28% discrepancy between the outcomes of non-invasive functional testing and invasive functional testing in adult patients with an interarterial and intraseptal course of AAOCA; (2) in 71% of these patients (20% of the overall cohort), invasive functional testing lead to an altered treatment recommendation, both more proactive surgical recommendations and recommendations of watchful waiting, compared with what the contemporary ESC and AHA/ACC guideline recommendation would dictate based on the real-world workup using non-invasive functional testing.

Current ESC and AHA/ACC guidelines base the risk stratification for SCD and treatment recommendations primarily on a few granular parameters: age, symptoms, anatomy, and non-invasive functional testing. While these parameters individually may be ambiguous, invasive functional testing has the potential to enhance risk stratification for SCD in individual AAOCA patients.

In clinical practice, age is an important and well-recognized discriminating factor for AAOCA management as it is particularly those under 35 years of age who are considered to be at increased risk for SCD.^4,5,14–16,20^ However, SCD still can occur in patients over 35 years, making age a less discriminative factor for assessing the risk in individual patients.^14–16^ The current cohort had a median age of 53 years [IQR 44–59]. Nevertheless, while SCD due to AAOCA later in life can occur, the risk of SCD should still be evaluated. Furthermore, it can be anticipated that AAOCA will increasingly be diagnosed in the adult population in the coming decades as the role of CTA in the diagnostic work-up of patients continues to expand, and CTA becomes more widely accessible.^42^ This projected increase in recognition and prevalence of AAOCA underlines the importance of individualized risk stratification and patient-tailored management strategies for adult AAOCA patients.

In line with previous literature, the current cohort demonstrated limited correlation between symptoms and the haemodynamic relevance of AAOCA, with only 21% initially presenting with cardiac pain in accordance with the ESC guidelines.^17,29^ The often non-anginal character of the chest pain in the majority of the patients often raises the question as to whether the finding of an AAOCA holds a causal relation with the complaints or should be regarded as an incidental finding. In particularly in patients of 50 years and above, other cardiac causes of cardiac chest pain should be considered—such as epicardial coronary artery disease or microvascular dysfunction.^43,44^

Likewise, risk stratification based primarily on high-risk anatomical features is ambiguous. In the current era, the identification of a high-risk anatomy AAOCA should prompt a comprehensive additional evaluation, particularly when surgical correction is being considered in accordance with established guidelines. Ideally, a multifactorial personalized risk stratification model should be developed, utilizing lesion specific cut-offs that are correlated to clinical outcomes. This is especially pertinent given our earlier study indicating that high-risk anatomy can be present even in the absence of positive results from both non-invasive and invasive functional testing.^20^ This suggests that high-risk anatomy may not necessarily lead to significant coronary flow obstruction. The current study confirms the highly variable utilization of various non-invasive functional testing modalities and protocols, *Table 3*, often dependent on local and regional expertise and technical availability.^21^ Of interest, despite the culminating evidence for the use of exertion or (if not possible) dobutamine or adrenaline for functional testing to best mimic the hyperdynamic setting of vigorous exercise, adenosine or regadenoson driven hyperaemia is still used (7% of the cohort).^21^ These pharmacological agents do not simulate the haemodynamic changes observed during physical exercise sufficiently and may further contribute to the poor predictive value of non-invasive functional testing in AAOCA patients.^22^ Invasive functional testing evaluates the haemodynamic consequences of AAOCA and is performed with pharmacological stress, e.g. dobutamine or adrenaline, which is believed to more accurately simulate the haemodynamic conditions encountered during physical exercise, potentially explaining the demonstrated incongruities.^22^

Notably, substantial discrepancies were also observed between non-invasive functional testing based on physical exercise and invasive functional testing in the current study. Among the patients who underwent physical exercise-based non-invasive functional testing, 7 (88%) patients with seemingly positive ischaemia on physical exercise testing results had negative outcomes when subjected to more rigorous invasive functional testing. These findings raise concerns on the predictive value of the non-invasive functional testing in the work-up of AAOCA, or at least questions the aetiology of the observed ischaemia in the non-invasive functional testing.

Although reflective of clinical practice, the heterogeneity of non-invasive functional testing modalities contributes to discrepant results compared with invasive testing, as diagnostic accuracy varies substantially between modalities. No non-invasive test has been established as a definitive gold standard in AAOCA; however, CMR and PET-CT are generally considered superior to SPECT and stress echocardiography for detecting subtle or regional ischaemia.^22,45,46^ In the present study, SPECT was predominantly utilized, likely due to institutional protocols and the relative accessibility to SPECT imaging within the clinical setting. Overall, non-invasive testing is characterized by higher specificity and lower sensitivity, which mainly result in false-negative findings.^22,47^ These limitations highlight the need for a multimodality approach, including invasive functional testing. Interestingly, recent studies have reported correlations between CCTA-derived parameters and dobutamine-based invasive FFR.^48^ Further research in this area may contribute to the development of a more robust and predominantly non-invasive diagnostic strategy in a subgroup of AAOCA patients. In the current study, the treatment recommendation was altered in 71% of the patients with discrepant outcomes of non-invasive and invasive functional testing, according to both ESC and AHA/ACC guidelines. Specifically, a substantial change in treatment recommendations—such as a shift from a surgical treatment recommendation (class I, I/IIa, or IIa) to conservative treatment (class IIb, IIb/III, or III), or vice versa—was observed in 71% of the cases according to the ESC guidelines and 57% of cases according to the AHA/ACC guidelines. In comparison to non-invasive functional testing, invasive functional testing, with its high spatial and temporal resolution, allows for the assessment of both the fixed and dynamic components of AAOCA when combined with appropriate pharmacological stress.^28^ This suggests that invasive functional testing may indeed be conceptually superior to non-invasive functional testing. Therefore, invasive functional testing may hold decisive clinical significance for treatment recommendations. However, prospective studies with long follow-up are warranted to evaluate the clinical outcomes, such as all-cause mortality, myocardial infarction, heart failure, and other relevant events, in patients managed based on invasive functional testing and to understand whether this strategy is indeed superior to the one currently advocated by the guidelines.

Of note, we were able to identify three distinct subgroups of AAOCA patients, accounting for 28% of the study population, who currently fall outside (i.e. no recommendation given by) the current ESC guidelines. These are defined as: (i) symptomatic (other than from typical angina or cardiac chest pain), patients with high-risk anatomy and proven ischaemia, (ii) asymptomatic AAORCA patients with high-risk anatomy, but without proven ischaemia, and (iii) asymptomatic AAOLCA patients ≥35 years of age without proven ischaemia and without high-risk anatomy. Recognition of these patient endotypes and their inclusion in future studies and ultimately revised guidelines is a necessity for inclusive and personalized care for AAOCA patients.

Based on the findings of this study, we recommend that diagnostic work-up for patients with high-risk anatomical features on CTA include invasive functional testing. Stress protocols should employ pharmacological agents, such as dobutamine or adrenaline, to replicate physical stress. The diagnostic accuracy of non-invasive tests is uncertain, whereas invasive functional testing provides a more detailed assessment of both anatomical and haemodynamic features in AAOCA. This recommendation is consistent with the current literature, including the study by Bigler *et al.*, which suggests that non-invasive testing may be considered optional for patients with right AAOCA and high-risk anatomy.^47^

### Limitations and future perspectives

This prospective multicentre study offers unique insights into the current state of practice and the intercentre variability in the utilization of the (non-) invasive functional testing protocols, however, it also introduces a degree of heterogeneity, with variations in diagnostic accuracies, the impact of which should be the focus of further studies. The results of this study should be interpreted with the consideration that it reflects clinical practice, with participant inclusion carried out according to a dedicated protocol/care pathway (MuSCAT). Additionally, this study did not assess the impact of discrepant outcomes between non-invasive and invasive testing on clinical outcomes, highlighting the need for further investigation in this area. Moreover, these data contribute to the understanding of invasive functional testing; however, further studies are needed to identify which patients benefit from additional invasive functional testing, for example by evaluating predictors from non-invasive diagnostics such as CTA. Although data on, or self-reported measures of, activity level contribute to individual risk stratification, this information is not reported in the present study due to its unavailability. The number of included patients is limited, inherent to the rarity of the condition, making this multicentre study one of the largest to date with paired invasive and non-invasive testing that reflects a contemporary cohort.

## Conclusions

This first prospective multicentre study on coronary anomalies in the Netherlands (MuSCAT study) in adult AAOCA patients revealed a 28% discrepancy between non-invasive and invasive functional testing, leading to altered management recommendations for 71% of these (20% of the total cohort) patients. Implementing optimal functional testing modalities may enhance risk stratification for ischaemic events and SCD and enables tailored treatment strategies for AAOCA patients. Further prospective research with long-term follow-up is needed to evaluate clinical outcomes.

## Supplementary Material

qyag010_Supplementary_Data

## Data Availability

Data are available upon reasonable request.

## Lead author biography

Mr Diederick Verheijen is a resident in cardiology and a physician-scientist with a clinical and research focus on congenital heart disease. His research primarily investigates anomalous coronary arteries and their clinical implications, with the aim of improving diagnostic accuracy and patient outcomes. Mr Diederick Verheijen combines clinical training with translational research to bridge the gap between imaging findings and surgical decision-making in congenital cardiac anomalies.
