# Supplementary material for: Adults with anomalous aortic origin of a coronary artery: impact of invasive functional testing on clinical decision making—insights from the MuSCAT registry
Source: Eur Heart J Imaging Methods Pract. 2026 Jan 17;3(3):qyag010. doi: 10.1093/ehjimp/qyag010 (PMC12931452; doi:10.1093/ehjimp/qyag010)
Supplement: qyag010_Supplementary_Data [file qyag010_supplementary_data.docx]

**Supplemental material**

**Figure S1** Number of patients included per participating center.

**Figure S2** Figure illustrating the coronary artery anatomy of the most prevalent AAOCA variants identified in this study, with corresponding nomenclature based on the Leiden Convention coronary coding system.

**Figure S3** UpSet plot illustrating the overlap of positive and negative findings for high-risk coronary anatomy, non-invasive functional testing, invasive functional assessment (FFR/iFR/RFR), and functional anatomical compression on IVUS among all patients with at least one positive result in non-invasive testing, invasive functional assessment, or IVUS**.**

**Figure S4** Bar graph illustrating the number of patients with negative, positive, and non-conclusive outcomes for the non-invasive functional testing and invasive functional testing.

**Table S1** Local MuSCAT center protocols for invasive functional testing in AAOCA.

**Supplemental material.**

**Figure S1** Number of patients included per participating center.


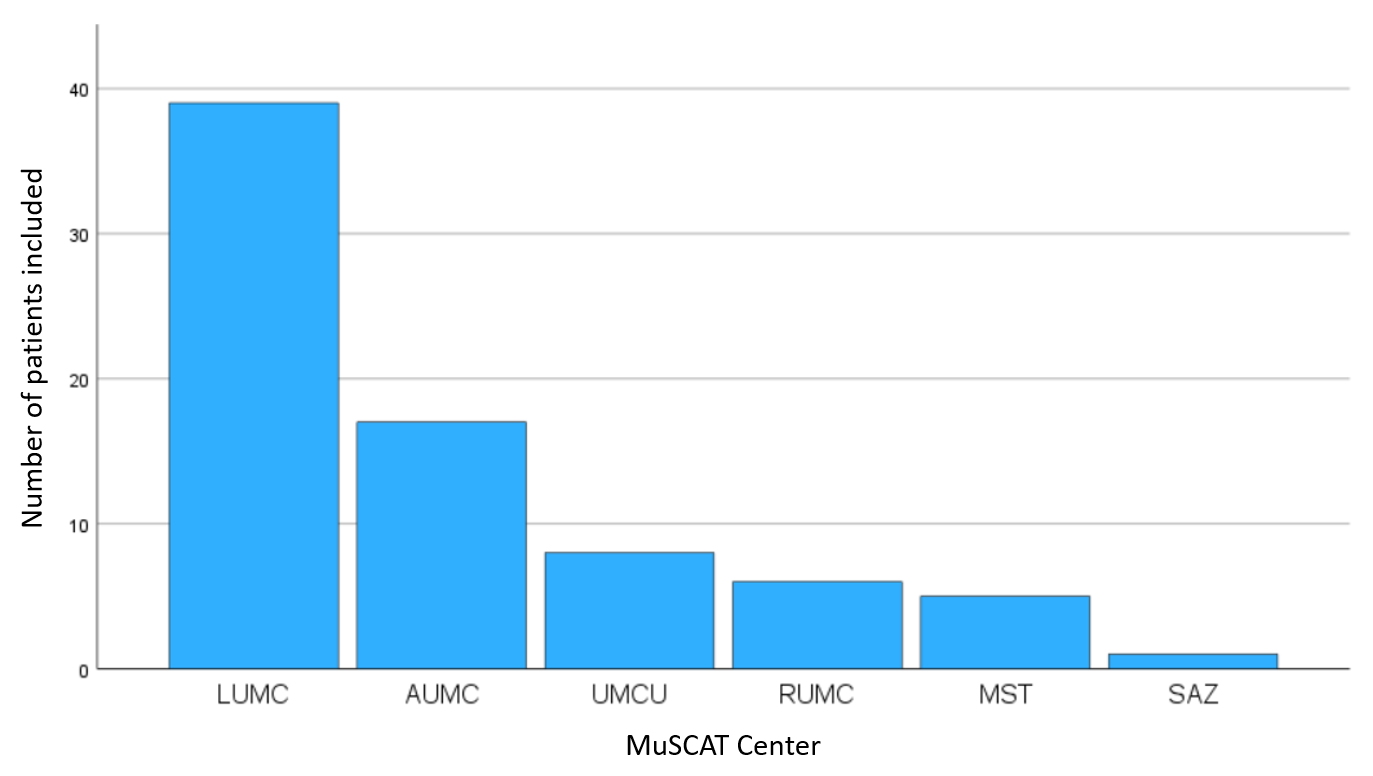


Abbreviations: AUMC, Amsterdam University Medical Center; LUMC, Leiden University Medical Center; RUMC, MST, Medisch Spectum Twente; Radboud University Medical Center; SAZ, St. Antonius Ziekenhuis; UMCU, University Medical Center Utrecht.

**Figure S2** Figure illustrating the coronary artery anatomy of the most prevalent AAOCA variants identified in this study, with corresponding nomenclature based on the Leiden Convention coronary coding system.


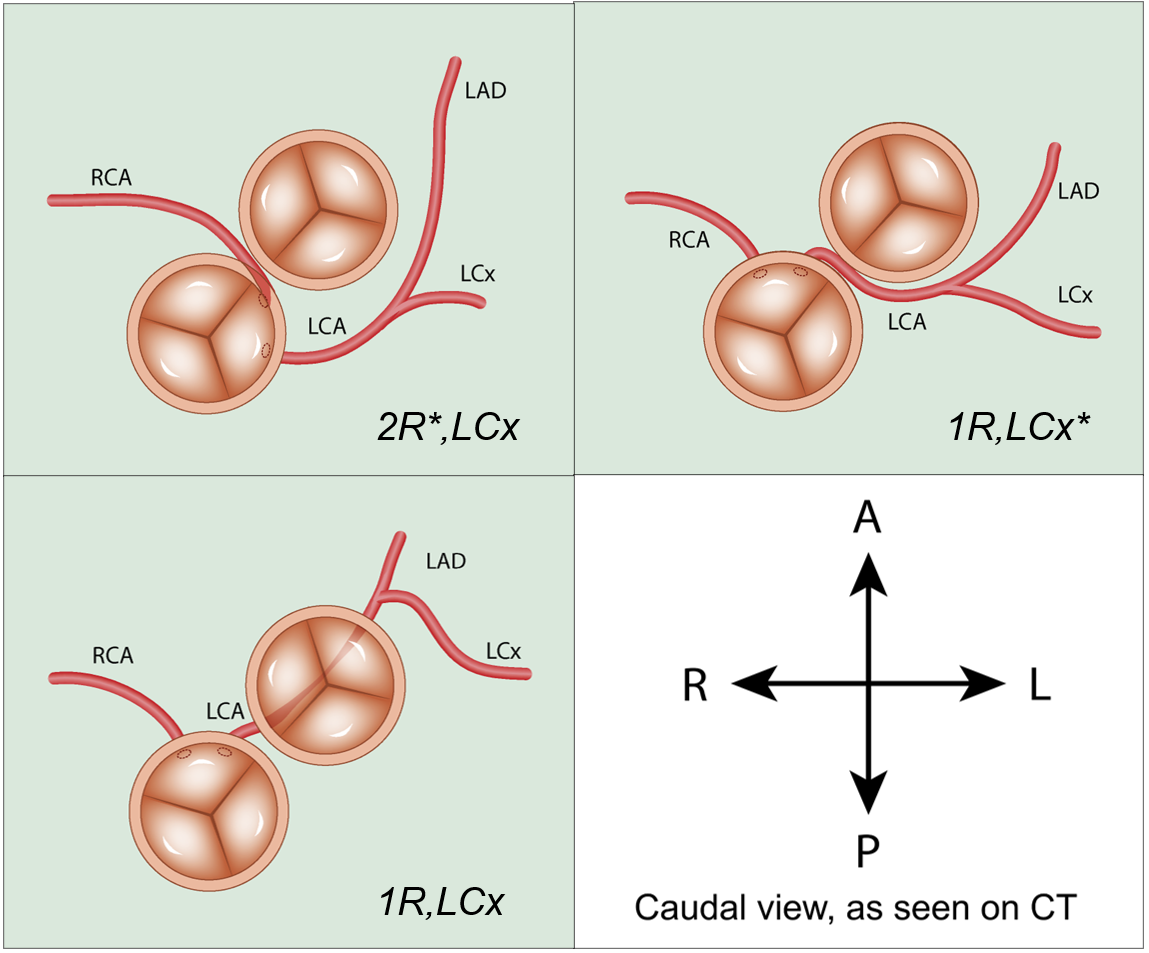


**Figure S3** UpSet plot illustrating the overlap of positive and negative findings for high-risk coronary anatomy, non-invasive functional testing, invasive functional assessment (FFR/iFR/RFR), and functional anatomical compression on IVUS among all patients with at least one positive result in non-invasive testing, invasive functional assessment, or IVUS**.**


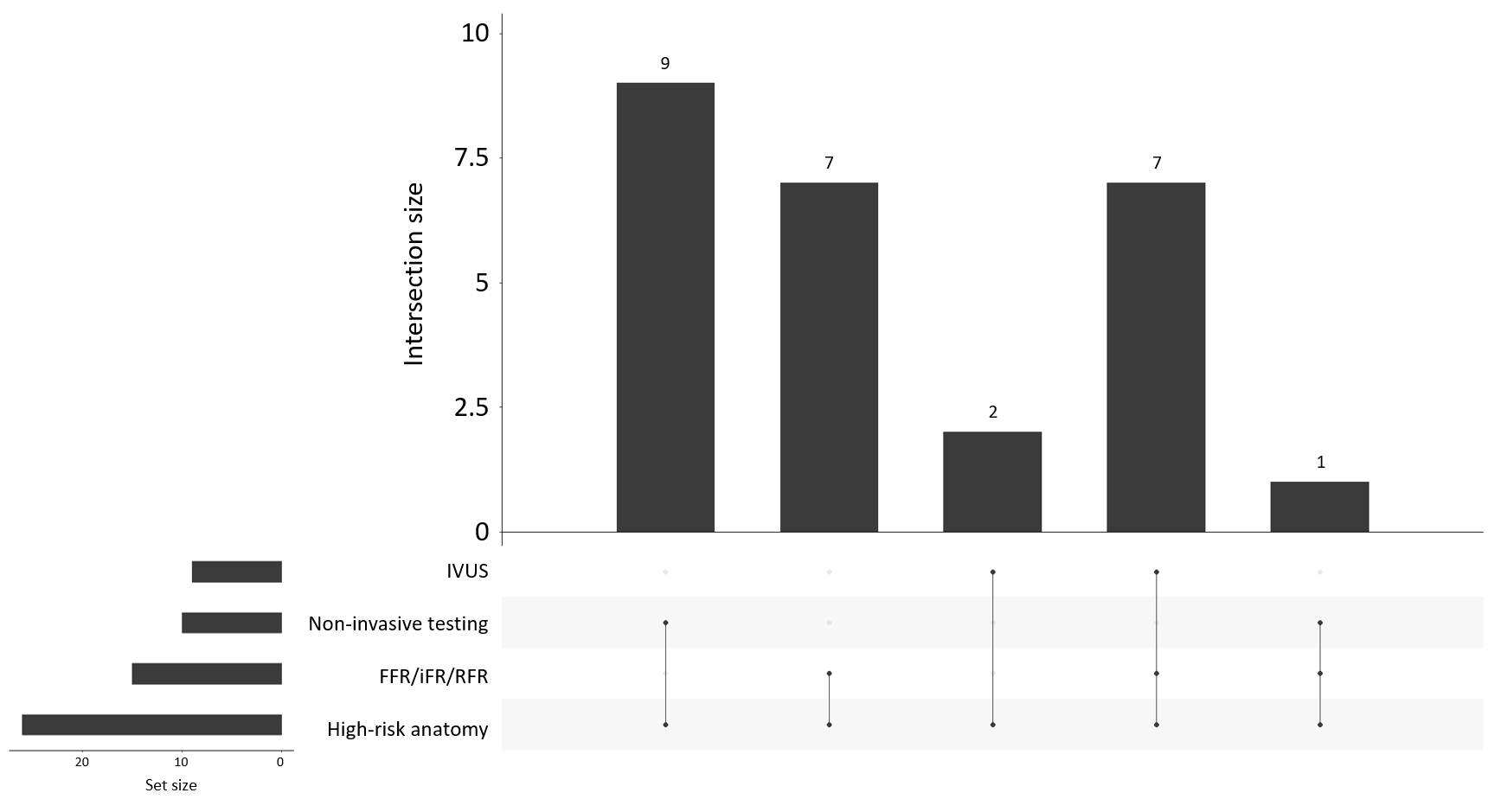


**Figure S4** Bar graph illustrating the number of patients with negative, positive, and non-conclusive outcomes for the non-invasive functional testing and invasive functional testing.


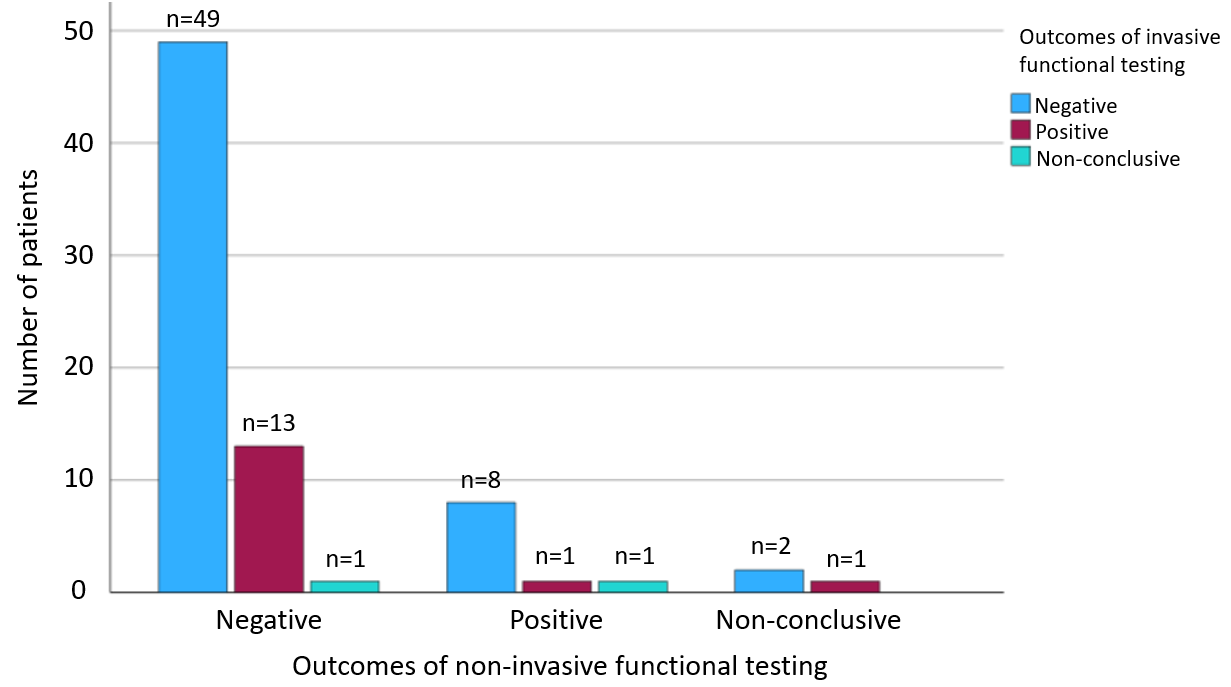


**Table S1** Local MuSCAT center protocols for invasive functional testing in AAOCA.

| **Site** | **Invasive testing with the cut-off values for a positive test** |
| --- | --- |
| LUMC | FFR: ≤0.80  iFR: ≤0.89  IVUS (slitlike orifice): ‘Lumen width’/’lumen height’ = ≤0.5  IVUS (dynamic compression): Decrease of ‘Lumen width’/’lumen height’ during pharmacological stress compared to baseline.  Pharmacological stress: Adrenaline |
| AUMC | FFR: ≤0.80  iFR: ≤0.89  IVUS (slitlike orifice): ‘Lumen width’/’lumen height’ = ≤0.5  IVUS (dynamic compression): Decrease of ‘Lumen width’/’lumen height’ during pharmacological stress compared to baseline.  Pharmacological stress: Dobutamine (with additional atropine (0,5mg) or handgrip) |
| UMCU | FFR: ≤0.80  iFR: ≤0.89  IVUS (slitlike orifice): ‘Lumen width’/’lumen height’ = ≤0.5  IVUS (dynamic compression): Decrease of ‘Lumen width’/’lumen height’ during pharmacological stress  Pharmacological stress: Dobutamine |
| RUMC | FFR: ≤0.80  RFR: ≤0.89  IVUS (slitlike orifice): ‘Lumen width’/’lumen height’ = ≤0.5  IVUS (dynamic compression): Decrease of ‘Lumen width’/’lumen height’ during pharmacological stress compared to baseline.  Pharmacological stress: Dobutamine |
| MST | FFR: ≤0.80  iFR: ≤0.89  IVUS (slitlike orifice): ‘Lumen width’/’lumen height’ = ≤0.5  IVUS (dynamic compression): Decrease of ‘Lumen width’/’lumen height’ during pharmacological stress compared to baseline.  Pharmacological stress: Dobutamine |
| SAZ | FFR: ≤0.80  Pharmacological stress: Dobutamine |

Abbreviations: AAOCA, anomalous aortic origin of a coronary artery; AUMC, Amsterdam University Medical Center; FFR, fractional flow reserve; iFR, instantaneous wave-free ratio; LUMC, Leiden University Medical Center; RUMC, MST, Medisch Spectum Twente; Radboud University Medical Center; RFR, resting full-cycle ratio; SAZ, St. Antonius Ziekenhuis; UMCU, University Medical Center Utrecht.
